# Construction and external validation of a 5-gene random forest model to diagnose non-obstructive azoospermia based on the single-cell RNA sequencing of testicular tissue

**DOI:** 10.18632/aging.203675

**Published:** 2021-11-04

**Authors:** Ranran Zhou, Xianyuan Lv, Tianle Chen, Qi Chen, Hu Tian, Cheng Yang, Wenbin Guo, Cundong Liu

**Affiliations:** 1 Department of Urology, The Third Affiliated Hospital of Southern Medical University, Guangzhou, China; 2 The Third School of Clinical Medicine, Southern Medical University, Guangzhou, China

**Keywords:** non-obstructive azoospermia, diagnosis, scRNA-seq, random forest, machine learning

## Abstract

Non-obstructive azoospermia (NOA) is among the most severe factors for male infertility, but our understandings of the latent biological mechanisms remain insufficient. The single-cell RNA sequencing (scRNA-seq) data of 432 testicular cells isolated from the patient with NOA was analyzed, and the cell samples were grouped into 5 cell clusters. A sum of 455 cell markers was identified and then included in the protein-protein interaction network. The Top 5 most critical genes in the network, including CCT8, CDC6, PSMD1, RPS4X, RPL36A, were selected for the diagnosis model construction through the random forest (RF). The RF model was a strong classifier for NOA and obstructive azoospermia (OA), which was validated in the training cohort (n = 58, AUC = 1) and external validation cohort (n = 20, AUC = 0.9). We collected the seminal plasma samples and testicular biopsy samples from 20 OA and 20 NOA cases from the local hospital, and the gene expression was detected via Real-Time quantitative Polymerase Chain Reaction (RT-qPCR) and Immunohistochemistry. The RF model also exhibited high accuracy (AUC = 0.725) in the local cohort. In summary, a novel gene signature was developed and externally validated based on scRNA-seq analysis, providing some new biomarkers to uncover the underlying mechanisms and a promising clinical tool for diagnosis in NOA.

## INTRODUCTION

Approximate 8-12% of couples suffered from infertility, and male infertility accounts for about 50% of the etiology therein [1]. Male infertility is mainly caused by impaired spermatogenesis, which is manifested clinically as azoospermia, oligospermia, teratozoospermia, and asthenospermia [2]. Azoospermia is the most severe factor of male infertility and included two major subtypes: obstructive azoospermia (OA) and non-obstructive azoospermia (NOA). The testes of patients with OA usually have normal sperm production ability, and abnormal sperm delivery due to obstruction results in azoospermia, while NOA is caused by impaired spermatogenesis in the testes, accounting for 10% of male infertility [3].

OA patients have normal spermatogenesis, but due to various pathological changes, such as seminal vesicle hypoplasia, chronic epididymitis, and prostatitis, the vas deferens obstruction prevents sperm from entering the semen [4]. The causes of NOA are more complicated. Common pathogenic factors include hereditary diseases, congenital testicular abnormalities, pathological changes of the testis, endocrine diseases, radiation, physical, chemical, and pharmaceutical damages [5]. Removing the obstruction of the vas deferens by microsurgery is the first choice for treatment of OA, while intracytoplasmic sperm injection (ICSI) and testicular sperm extraction (TESE) are more recommended for NOA [6]. Hence, the differential diagnosis of OA and NOA is of great significance because it is directly related to the choice of treatment methods [7].

With the rise and advancement of gene sequencing and big-data analysis, the development of gene-based models for disease diagnosis has attracted increasing attention. These genetic models acted as valuable tools to guide clinical practice and provided potential clues for investigating pathogenesis [8, 9]. Among the high-throughput sequencing methods, single-cell RNA sequencing analyzed the transcriptomics at single-cell resolution, assessing the cell heterogeneity and diversity with high efficacy [10]. The scRNA-seq-based models have been successfully established in various diseases, such as bladder cancer [11], pancreatic ductal adenocarcinoma [12], and skin cancer [13], showing the tremendous advantages of scRNA-seq to achieve greater understandings of disease initiation and progression. However, no genetic diagnostic model for NOA based on scRNA-seq has been constructed.

The present study analyzed the scRNA-seq data of 432 testicular cells isolated from the patient with NOA and screened the marker genes among different cell clusters. Subsequently, a protein-protein interaction (PPI) network was established, and the hub genes of the network were identified. The random forest algorithm was utilized for diagnostic model construction for NOA, and two independent NOA datasets were downloaded from Gene Expression Omnibus (GEO) as the training and external validation cohorts, respectively. The collected samples from The Third Affiliated Hospital of Southern Medical University were also utilized for validation through Real-Time quantitative Polymerase Chain Reaction (RT-qPCR).

## MATERIALS AND METHODS

### Data collection

The scRNA-seq matrix of 432 testicular cells from an NOA patient (GSE157421) was directly downloaded on GEO (https://www.ncbi.nlm.nih.gov/geo/). GSE9210, including 11 OA and 47 NOA samples, and GSE145467, including 10 OA and 10 NOA samples, were also obtained as the training and external validation datasets, respectively. GSE9210 and GSE145467 were both Agilent microarray experiments for human testicular tissues. The probe IDs were converted into gene symbols using R software (version 4.1.0).

### Processing of scRNA-seq data

The Seurat package of R was used to normalize the scRNA-seq data and to perform the quality control [14]. The filtering criteria were set as follows: nFeature_RNA > 50 and percent.mt < 5, which meant the cells with detected gene numbers ≤ 50 and the proportion of mitochondria ≥ 5% were excluded from the present study. The Top 10 genes exhibiting the most variable among the cell samples were identified with the FindVariableFeatures function of Seurat. Subsequently, the cell samples clustering was conducted via principal component analysis (PCA) and t-distributed stochastic neighbor embedding (t-SNE) based on the Top 1500 most variable genes. The markers genes of various cell clusters were screened with |logarithmic fold change [logFC]| > 0.8 and adjusted P < 0.05. The cell types were annotated via the SingleR and celldex packages. The monocle package was adopted for pseudotime analysis, which re-verified the correctness of the cell type annotation.

### Construction of the protein-protein interaction network

The cell markers extracted from the scRNA-seq analysis were then used to establish a protein-protein interaction (PPI) network to identify the possible hub genes associated with the pathogenesis of NOA through the STRING database (https://string-db.org/). The confidence score was set to 0.9 to ensure the reliability of the established network as possible. The cytoHubba plug-in of Cytoscape software (version 3.8.0) was used to measure the importance of the genes in the network via degree algorithm.

### Functional enrichment

Gene Ontology (GO) and Kyoto Encyclopedia of Genes and Genomes (KEGG) enrichment were conducted to annotate the biological functions of the genes in the network through the clusterProfiler R package. The gene sets with P < 0.05 and Q < 0.05 were considered to be statistically significant.

### Development of a random forest model

The Top 5 core genes in the network were chosen as the variables for model construction. The diagnostic model was developed through the randomForest R package. Ntree =500 and mtry=3 was set as the arguments for the random forest. Mtry is defined as the number of variables sampled per iteration and ntree refers to the number of decision trees contained in the random forest. The pROC package was utilized to draw the receiver operating curves (ROCs) and to evaluate the 95% confidence interval (CI) of areas under curves (AUCs) based on 2000 bootstrap sampling, which were applied to measure the random forest’s predictive performance in the training and external validation datasets. The confusion matrices were visualized via R software. Mean Decrease Accuracy and Mean Decrease Gini were used to calculate the importance of the variables in the random forest model, which were positively associated with the importance. Mean Decrease Accuracy meant the degree of reduction in the accuracy of random forest prediction after changing the value of a variable into a random number, and Mean Decrease Gini meant the influence of each variable on the heterogeneity of observations at each node of the classification tree [15]. We also compared the AUCs of the single gene and the 5-gene RF model via Delong’ test to check whether the AUCs have been significantly altered. The expression divergence of the hub genes in each cell cluster was visualized via a bubble plot and a scatter plot with the Seurat package.

### Clinical sample collection

This study protocol was approved by the Medical Ethics Committee of The Third Affiliated Hospital of Southern Medical University, and written consent was obtained from all patients. The patients with OA and NOA between October 2019 and September 2021 were enrolled in this study, and the diagnosis of OA or NOA relied on the testicular biopsy. The biopsy samples were immediately fixed with 4% paraformaldehyde (ThermoFisher Scientific, China) overnight, embedded in paraffin, and sectioned 8-10 μm thick. Age, Johnsen’s Score, follicle-stimulating hormone (FSH), luteinizing hormone (LH), and testosterone (T) of the cases were also collected.

All the study subjects were abstinent for 3-5 days before the semen collection. The semen samples were obtained by masturbation. The semen was liquified for 20-30 minutes at room temperature. The seminal plasma was collected by centrifuging the semen at 4° C at 10000 x g for 10 minutes, and the precipitate was discarded. The seminal plasma was stored at -80° C for further study.

### RT-qPCR

The total RNA was extracted with the Trizol-chloroform method (Trizol reagent, Invitrogen, USA) after keeping the seminal plasma gently thawed on ice. The cDNA was synthesized with PrimeScript RT Reagent Kit (Takara, China) and amplified by SYBR Premix ExTaq kit (Takara, China) following the manufacturer’s recommendations. The qualification of the RNA expression was based on ABI 7600 system (Applied Biosystems, USA). GAPDH was chosen as the internal reference gene. The 2−ΔΔCt methods were utilized to calculate the gene expression value. All the PCR experiments were repeated three times. The primer sequence was synthesized by the TSINGKE company (Guangzhou, China), as shown in Table 1.

**Table 1:** The primers used in present study for RT-qPCR.

**Genes**	**Sequence (5’-3’)**
CCT8: Forward	AGGAGGGAGCGAAACACTTTT
CCT8: Reverse	GTTGCTGCATCGTTTGTCACA
CDC6: Forward	CCAGGCACAGGCTACAATCAG
CDC6: Reverse	AACAGGTTACGGTTTGGACATT
PSMD1: Forward	TCCGAGTCCGTAGACAAAATAGA
PSMD1: Reverse	CCACACATTGTTTGGTGTAGTGA
RPL36A: Forward	CTAAAACCCGCCGGACTTTCT
RPL36A: Reverse	CTTCCTGTCATAACGCCGCTT
RPS4X: Forward	TGGCAGCTCCAAAGCATTG
RPS4X: Reverse	GACACTCTCTCAACTTGTGGG
GAPDH: Forward	GGAGCGAGATCCCTCCAAAAT
GAPDH: Reverse	GGCTGTTGTCATACTTCTCATGG

### Immunohistochemistry

The slides were washed with xylene and added to the ethanol as follows: 100% ethanol for 4 minutes; 90% ethanol for 4 minutes; 80% ethanol for 4minutes; 70% ethanol for 4 minutes. The sections were repaired in antigen repair solution (ThermoFisher Scientific, China) for 10 minutes at 95° C. 5% bovine serum albumin (BSA) in phosphate buffered saline (PBS) was used to block the non-specific binding sites for 1 hour. During the immunohistochemical staining with RPS4X (1:100, Proteintech, China), the slides and the antibody were incubated for 2 hours at room temperature in a humidified chamber. After the slides were washed 3 times with PBS, the anti-rabbit secondary antibody (Proteintech, China) was added to the slides for 1 hour at room temperature. Images were acquired by standard microscopy (Nikon Eclipse 90i, Nikon, Japan). The gray-scale of the images were analyzed according to the integral optical density (IOD), which was calculated by Image-Pro Plus (version 6.0, Media Cybernetics, USA).

### Statistical analysis

The statistical analyses were based on R software (version 4.0.3, R core team) and GraphPad Prism 8 (version 8.4.3, GraphPad, USA). All the data of this study was presented as mean ± standard deviation (SD). The two-tailed Student’s t-test was performed for the variance detection for the RT-qPCR and immunohistochemistry data, and P < 0.05 was considered as statistically significant. Welch-corrected t-test was utilized to compare the difference of age, Johnsen’s Score, FSH, TH, and T between OA and NOA cases. Delong’s test was conducted to compare the AUCs of different ROCs by means of roc.test function of R pROC package. *P < 0.05, **P < 0.01, ***P < 0.001.

## RESULTS

### Identification of cell markers via scRNA-seq analysis

**Figure 1:**
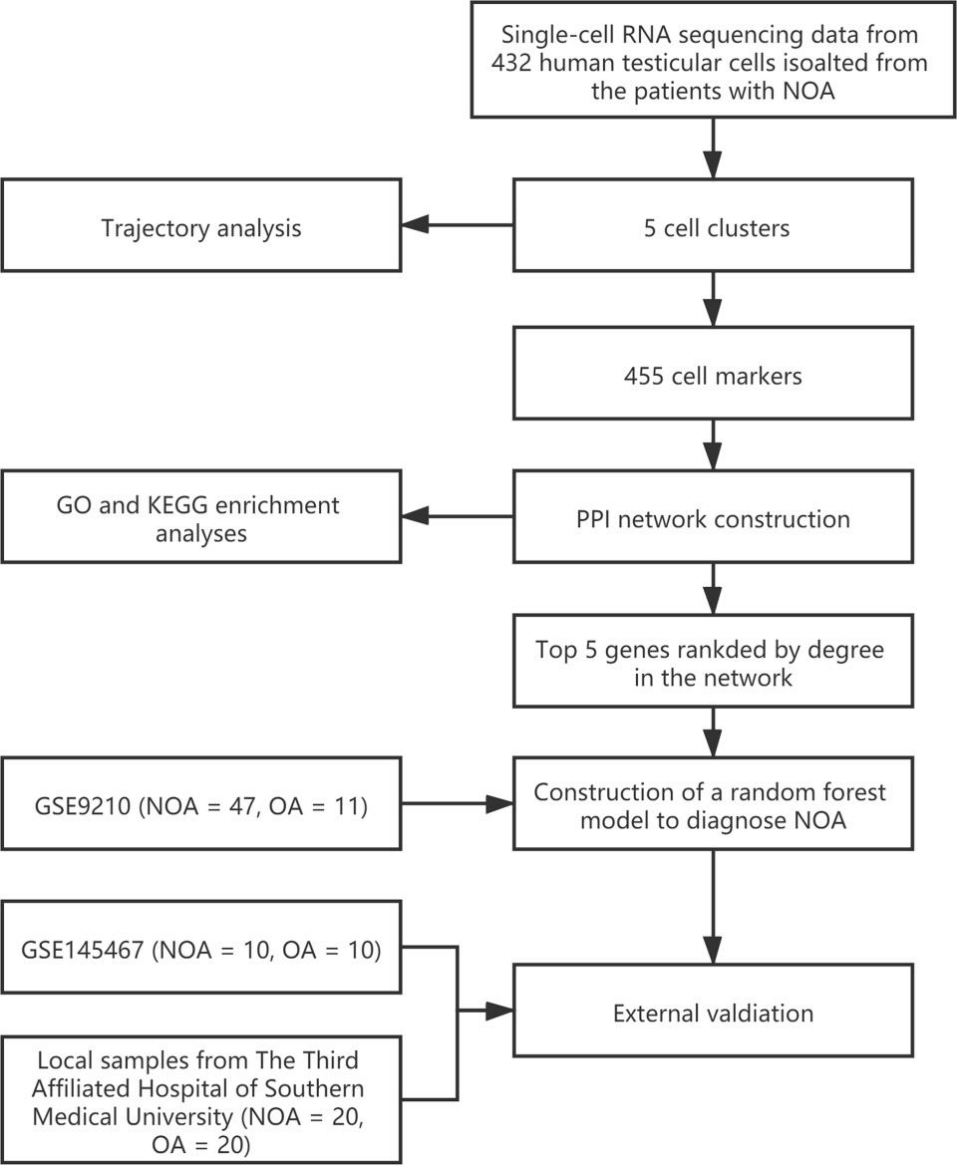
The workflow of the present study.

**Figure 2:**
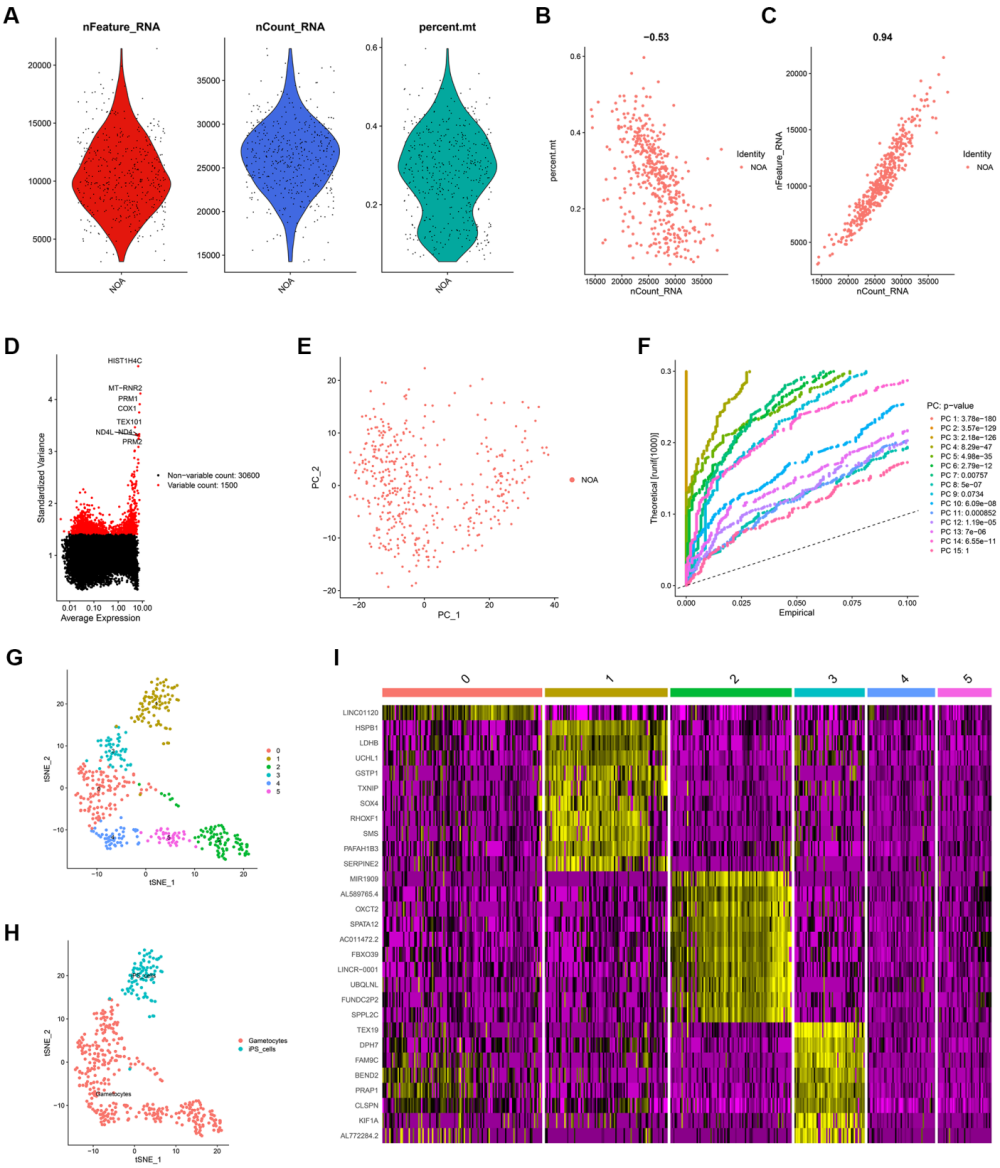
**The identification of cell markers via scRNA-seq analysis.** (**A**) The quality control chart. (**B**, **C**) The association of detected gene counts with the percent of mitochondrial genes (**B**) and sequencing depth (**C**). (**D**) The Top 10 genes with the most differentially expressed among various cell samples. (**E**) The PCA analysis. (**F**) The P-values of each PC. (**G**) The cell samples were divided into 5 clusters. (**H**) The cell type annotation. (**I**) The heat map indicating the expression level of the cell markers in different cell clusters. scRNA-seq, single-cell RNA sequencing; PCA, principal component analysis; PC, principal component.

The workflow of the present study was shown in Figure 1. First, the scRNA-seq data of an NOA patient’s testicular sample was analyzed, and a sum of 432 testicular cells was acquired. The quality control of the detected gene numbers, gene sequencing count, and the percent of mitochondrial genes was indicated in Figure 2A. The percent of mitochondrial genes was negatively associated with detected gene counts (Pearson r = -0.53, Figure 2B); meanwhile, the high positive correlation between sequencing depth and detected gene counts was found (Pearson r = 0.94, Figure 2C). The Top 10 genes, including HIST1H4C, MT-RNR2, PRM1, COX1, TEX101, ND4, ND4L, and PRM2, showing the most significant expression difference among all cell samples, were revealed in Figure 2D. Subsequently, PCA was conducted to preliminarily classify the cell samples (Figure 2E), and the P-value distribution in each principal component (PC) was shown in Figure 2F. The Top 20 and Top 30 genes associated with PC1-4 were illustrated in the dot plot (Supplementary Figure 1A) and the heat map (Supplementary Figure 1B), respectively. With the t-SNE dimension-reduction algorithm, 432 testicular cells were divided into 5 different cell clusters (Figure 2G). Cell cluster 1 was annotated as induced pluripotent stem (iPS) cells, and the remaining 4 cell clusters were all annotated as gametocytes (Figure 2H). Ultimately, a total of 456 cell markers were identified with the limma package (Supplementary Table 1). The heat map displayed the expression level of Top 10 differentially expressed genes (DEGs) among the cell clusters (Figure 2I).

### Cell trajectory analysis

**Figure 3:**
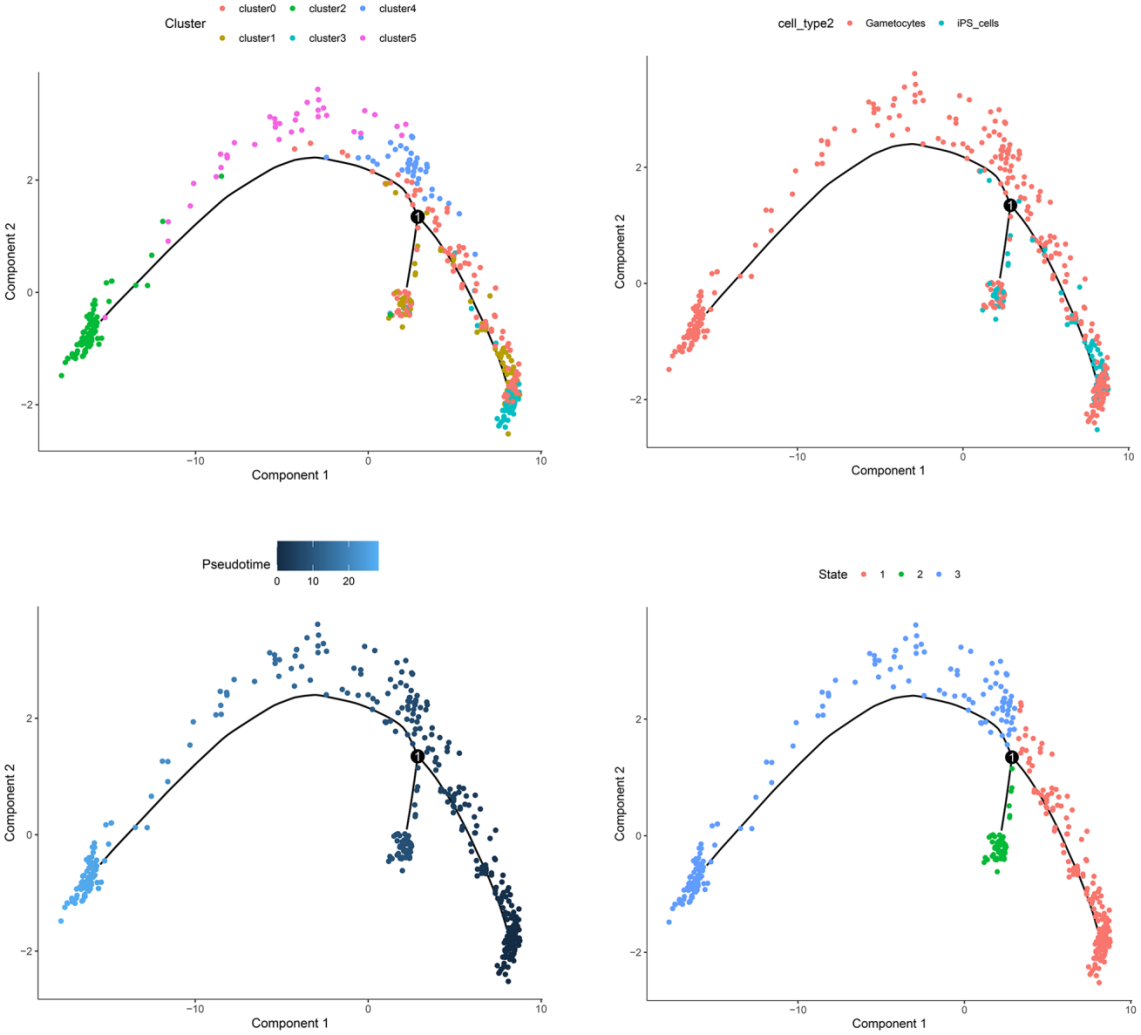
The trajectory analysis of the cell samples.

In scRNA-seq analysis, the correct cell type annotation has always been a difficult point. We conducted the pseudotime analysis to confirm whether the cell type annotation was right. As shown in Figure 3, iPS cells gradually differentiated into gametocytes over time, which was reasonable and logical. The cell trajectory analysis validated the annotation results.

### PPI network construction and functional enrichment

**Figure 4:**
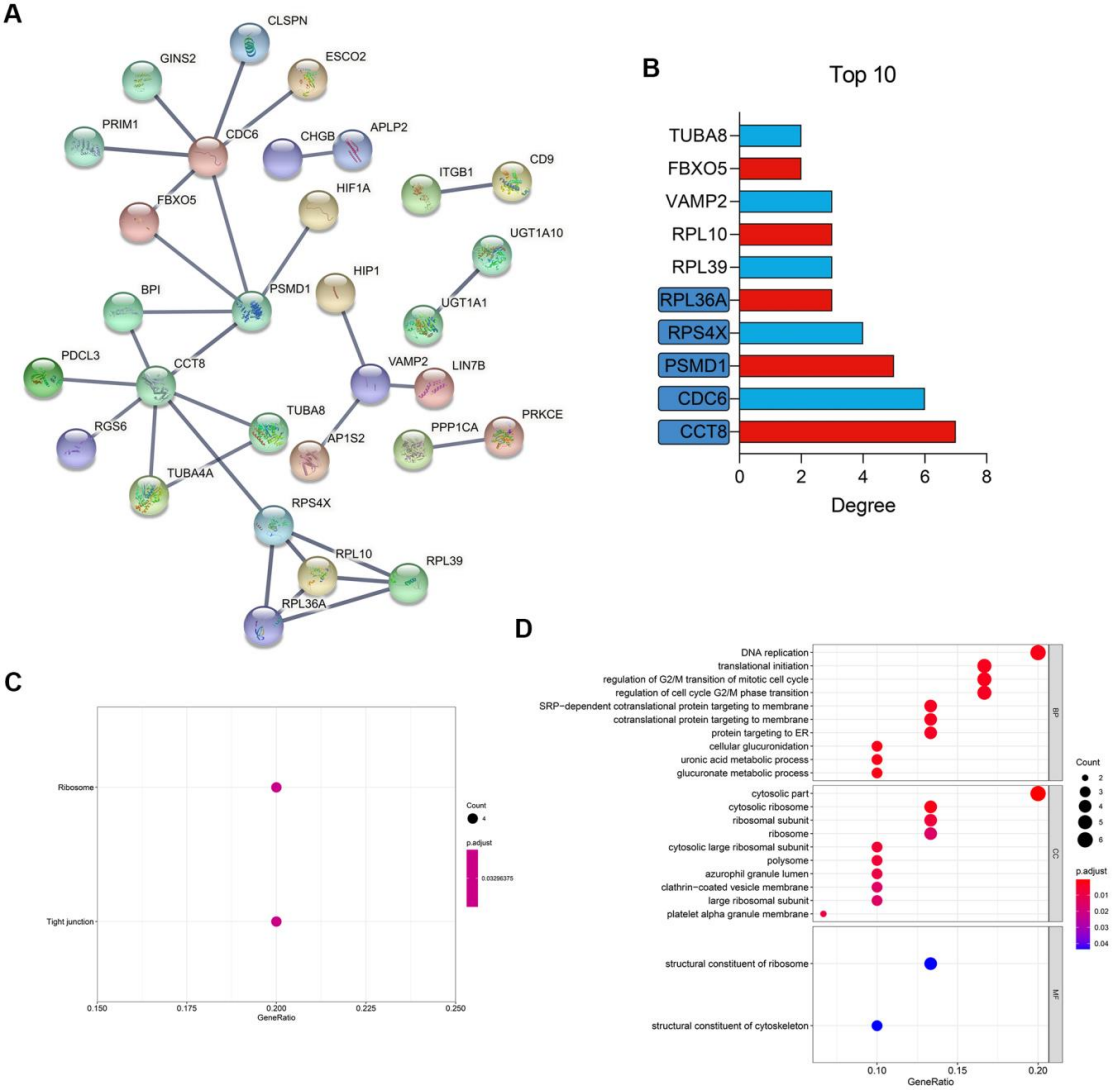
**PPI network construction and functional enrichment.** (**A**) Construction of a PPI network of the cell markers. (**B**) The Top 10 most important gene in the network. (**C**) KEGG pathway enrichment. (**D**) GO functional annotation. PPI, protein-protein interaction; KEGG, Kyoto Encyclopedia of Genes and Genomes; GO, Gene Ontology.

Compared with the DEGs extracted from the tissue with different statuses, such as OA and NOA, the DEGs between different cell clusters, also known as cell markers, could reflect the pathogenesis with a higher resolution. Hence, the cell markers from the scRNA-seq analysis were then used to construct the PPI network. With the confidence score > 0.9 filtering, 30 genes were included in the network, as displayed in Figure 4A. The Top 10 hub genes ranked by degree were shown in Figure 4B and Supplementary Table 2. KEGG (Figure 4C) and GO (Figure 4D) functional annotation indicated the genes in the PPI network were mostly enriched in ribosome, tight junction, DNA replication, and many other critical pathways involved in cell activities.

### Establishment and validation of a random forest model

**Figure 5:**
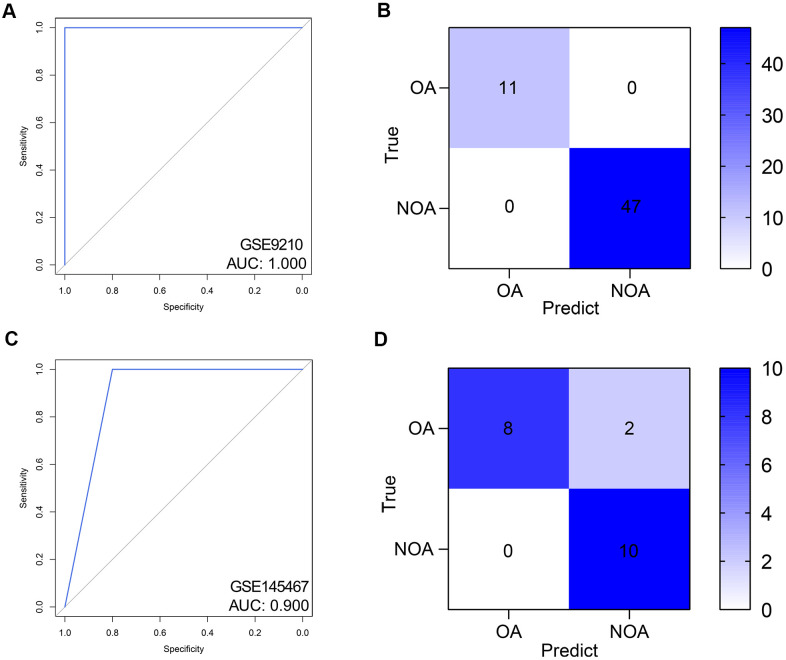
**Validation of the diagnostic efficacy of the random forest model.** (**A**, **B**) The ROC (**A**) and confusion matrix (**B**) of the predictive model in the training dataset. (**C**, **D**) The ROC (**C**) and confusion matrix (**D**) of the predictive model in the external validation dataset. ROC, receiver operating curve. AUC, area under curve; NOA, non-obstructive azoospermia; OA, obstructive azoospermia.

Here, we detected the diagnostic value of the hub genes of NOA from the scRNA-seq and PPI analyses. Compared with the single gene, a multi-gene combination would be more potent for prediction, which has been demonstrated in many previous studies [16]. With the rapid development of computer technology, machine learning is increasingly applied to disease diagnosis [17, 18]. Hence, we implemented random forest, a widely used and powerful machine learning algorithm, to construct the diagnosis model [19, 20]. GSE9210 was set as the training dataset, and AUC of the random forest model was 1.000 (95% CI = 1.000-1.000), as displayed in the ROC (Figure 5A) and confusion matrix (Figure 5B). The performance of the diagnostic model in the external validation was also favorable with the AUC = 0.900 (95% CI = 0.769-1.000). Figure 5C, 5D showed the ROC and confusion matrix of the established model in the external validation cohort.

**Figure 6:**
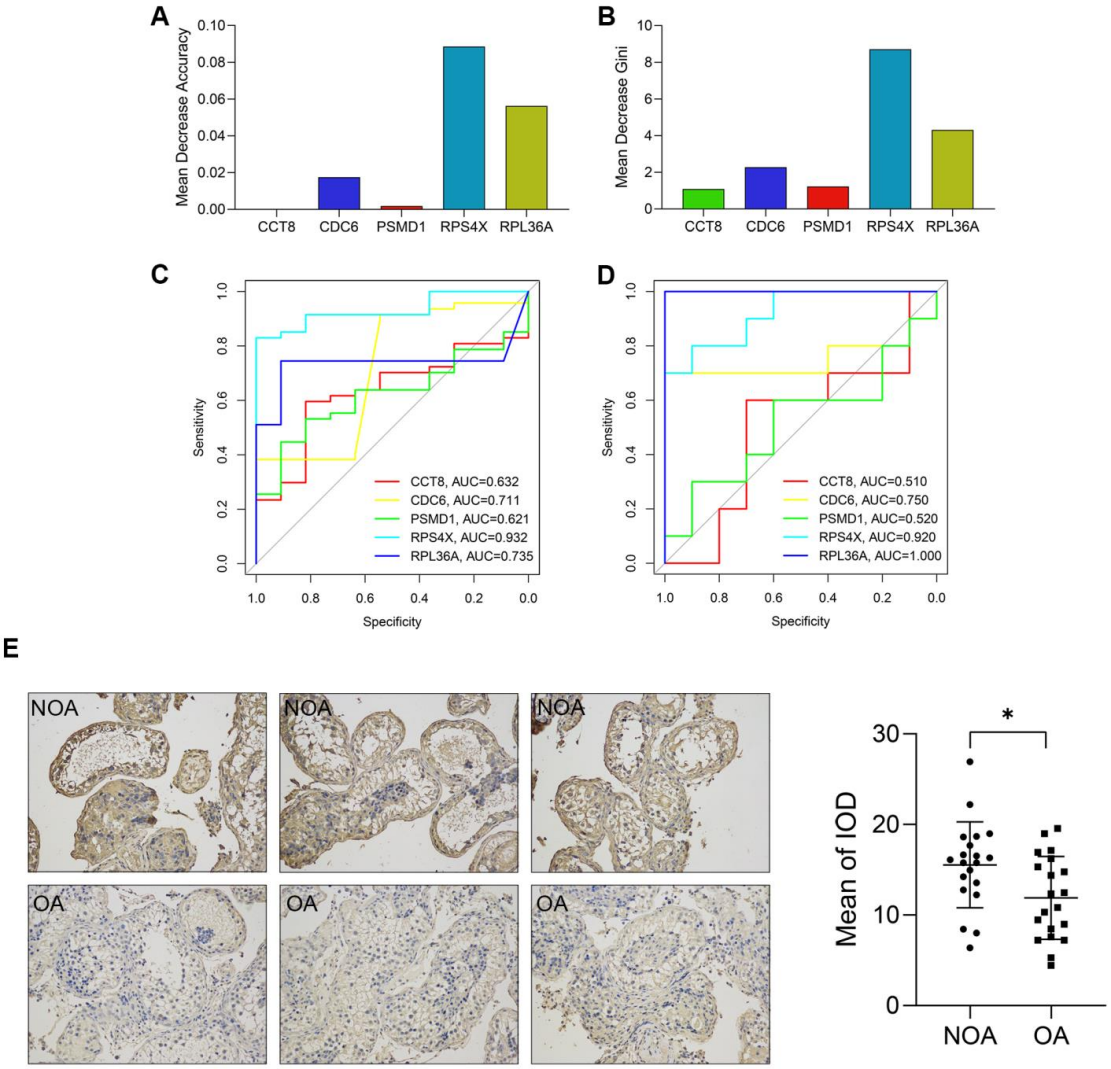
**The diagnostic value of each variable in the random forest model.** (**A**, **B**) The Mean Decrease Accuracy (**A**) and Mean Decrease Gini (**B**) of the variables. (**C**, **D**) The ROCs showed the predictive performance of each gene in the training (**C**) and external validation cohorts (**D**). (**E**) The expression of RPS4X in the testicular biopsy samples from 20 NOA (up) and 20 OA (down) patients (x200). ROC, receiver operating curve. AUC, area under curve; NOA, non-obstructive azoospermia; OA, obstructive azoospermia; IOD, integral optical density. *, P < 0.05.

The diagnostic value of the genes in the diagnostic model was also detected. First, the Mean Decrease Accuracy (Figure 6A) and Mean Decrease Gini (Figure 6B) of each gene were calculated, and RPS4X was found to serve as the most important variables in the random forest model. Besides, ROC analyses indicated the AUCs of RPS4X were 0.932 and 0.920 in the training and external validation datasets, respectively, suggesting RPS4X was a promising biomarker for NOA (Figure 6C, 6D). In addition, Delong’s test between the AUCs of the 5-gene RF model and the single gene indicated that RPS4X and RPL36A were important variables in the model, as displayed in Table 2.

**Table 2:** P-values of the Delong’s tests.

**The comparison**	**GSE9210**	**GSE145467**	**Local cohort**
CCT8 vs. The RF model	< 0.001	< 0.001	0.941
CDC6 vs. The RF model	0.002	0.313	0.442
PSMD1 vs. The RF model	< 0.001	0.007	0.692
RPS4X vs. The RF model	0.035	0.756	0.047
RPL36A vs. The RF model	<0.001	0.134	0.796

ROC, receiver’s operating curve; RF, random forest.

The expression level of RPS4X in human testicular samples from NOA and OA patients was also detected via immunohistochemical staining. It was found that RPS4X was significantly up-regulated in the testes of 20 NOA patients compared with that in 20 OA patients, as displayed in Figure 6E.

**Figure 7:**
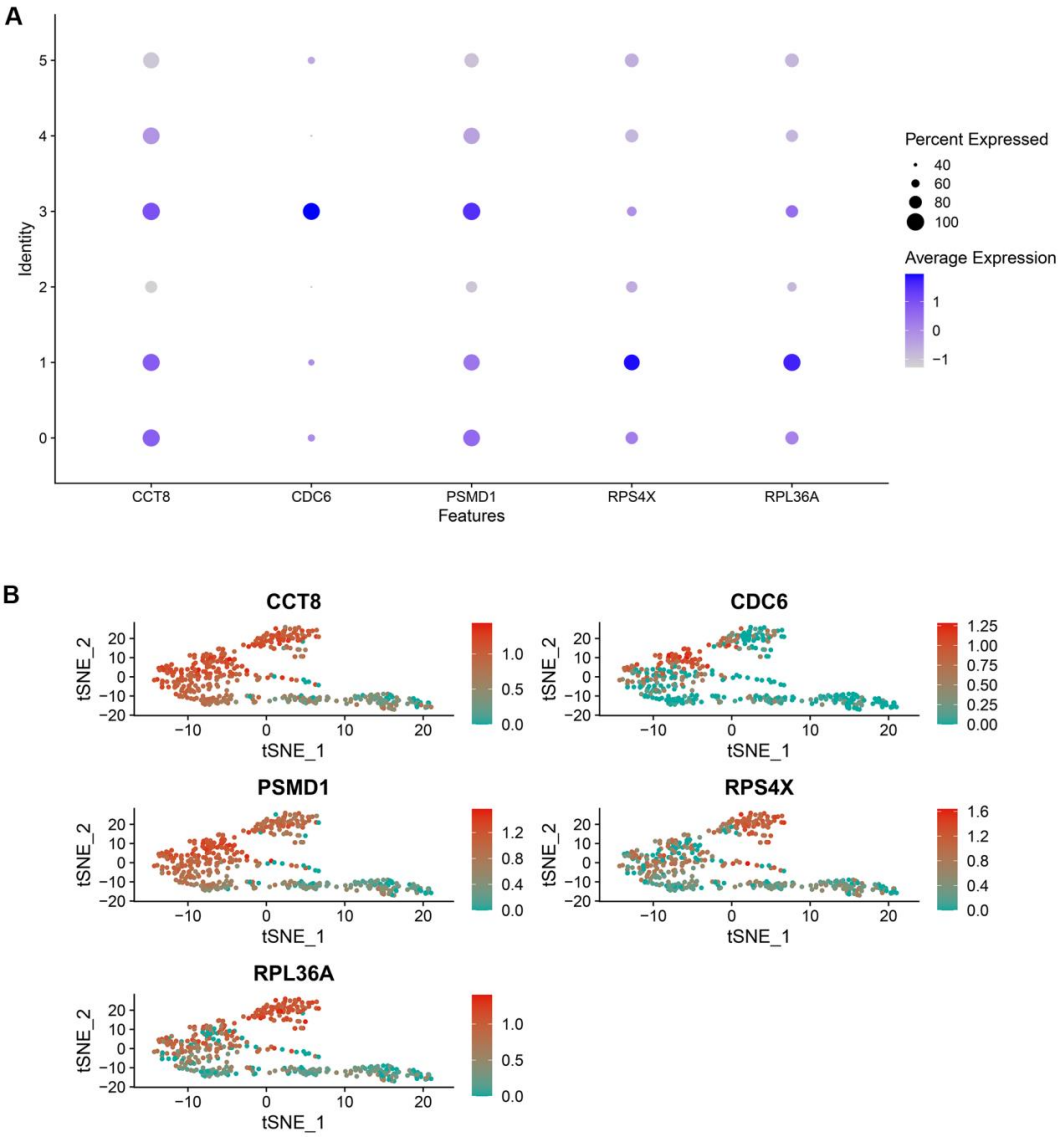
The expression level of the random forest model’s genes in each cell cluster, which was visualized by a bubble plot (**A**) and a scatter diagram (**B**).

In addition, the expression level of the hub genes in the cell clusters was also compared. As indicated in Figure 7A, 7B, RPS4X was significantly up-regulated in cell cluster 1, which was annotated with iPS cells, implying RPS4X might exert their pathogenetic functions during the differentiation of iPS cells into gametocytes.

### Experimental validation of the RF model in seminal plasma samples

**Figure 8:**
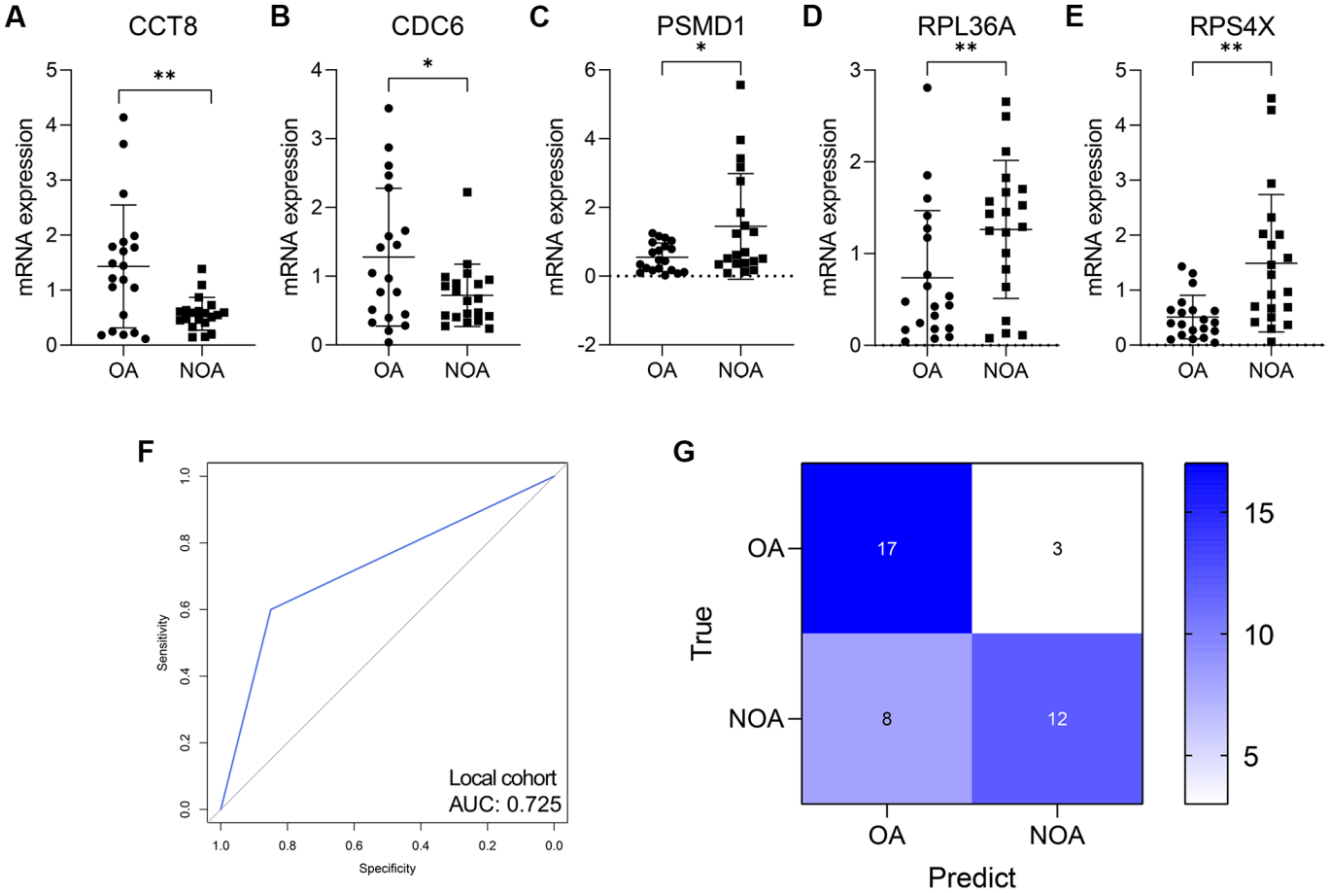
**The validation in human seminal plasma samples.** (**A**–**E**) The expression level of CCT8 (**A**), CDC6 (**B**), PSMD1 (**C**), RPL36A (**D**), and RPS4X (**E**) in human seminal plasma from 20 OA and 20 NOA patients. (**F**, **G**) The ROC (**F**) and confusion matrix (**G**) displayed that the RF model was a promising classifier in the collected human samples. NOA, non-obstructive azoospermia; OA, obstructive azoospermia. *, P < 0.05; **, P < 0.01; ***, P < 0.001.

A total of 40 azoospermia patients were enrolled, containing 20 OA and 20 NOA subjects, after excluding the samples with low RNA’s amount and unclear diagnosis. The clinical characteristics of the subjects were displayed in Table 3. The merge of multiple groups with different means and SDs from GSE9210 cohort into one group was conducted via an online tool (http://www.obg.cuhk.edu.hk/ResearchSupport/StatTools/CombineMeansSDs_Pgm.php). We analyzed the mRNA expression level of CCT8, CDC6, PSMD1, RPL36A, and RPS4X, which comprised the RF model, in the seminal plasma of OA and NOA patients. It was found that CCT8 (P < 0.01, Figure 8A) and CDC6 (P < 0.05, Figure 8B) were significantly up-regulated in the OA samples, while PSMD1 (P < 0.05, Figure 8C), RPL36A (P < 0.01, Figure 8D), and RPX4X (P < 0.05, Figure 8E) were obviously decreased in NOA patients’ seminal plasma. Table 4 indicated the AUCs and corresponding 95% CI of each gene in GSE9210 cohort, GSE145467 cohort, and local cohort.

**Table 3:** The baseline information of the OA and NOA patients from GSE9210 cohort and local cohort.

**Parameters**	**GSE9210**			**Local cohort**		
	**OA (n = 11)**	**NOA (n = 47)**	**P-value**	**OA (n = 20)**	**NOA (n = 20)**	**P-value**
Age (years)	33.3 ± 8.5	35.0 ± 5.7	0.542	32.5 ± 6.7	34.1 ± 7.4	0.478
Johnsen's Score	7.9 ± 1.2	2.4 ± 1.3	< 0.001	7.7 ± 1.5	3.5 ± 0.9	< 0.001
FSH (mIU/ml)	10.1 ± 9.3	29.2 ± 9.1	< 0.001	11.2 ± 8.8	23.8 ± 9.5	< 0.001
LH (mIU/ml)	4.5 ± 2.3	8.8 ± 4.8	< 0.001	5.3 ± 2.6	7.5 ± 2.2	< 0.01
T (ng/ml)	4.8 ± 1.7	3.5 ± 1.6	0.041	4.2 ± 1.1	3.6 ± 0.7	0.043

FSH, follicle-stimulating hormone; LH, luteinizing hormone; T, testosterone; NOA, non-obstructive azoospermia; OA, obstructive azoospermia.

**Table 4:** The AUCs of the genes and the RF model in each cohort.

**ID**	**GSE9210**		**GSE145467**		**Local cohort**	
	**AUC**	**95% CI**	**AUC**	**95% CI**	**AUC**	**95% CI**
CCT8	0.632	0.475-0.790	0.510	0.231-0.789	0.734	0.556-0.912
CDC6	0.711	0.532-0.889	0.750	0.489-1.000	0.640	0.456-0.824
PSMD1	0.621	0.471-0.771	0.520	0.247-0.793	0.679	0.508-0.850
RPS4X	0.932	0.869-0.995	0.920	0.805-1.000	0.798	0.656-0.939
RPL36A	0.735	0.608-0.862	1.000	1.000-1.000	0.700	0.528-0.872
The RF model	1.000	1.000-1.000	0.900	0.769-1.000	0.725	0.589-0.861

AUC, area under curve; CI, confidence interval; RF random forest.

The RF model was also a promising classifier in the seminal plasma from the ROC analysis (AUC = 0.725, 95% CI = 0.589-0.861, Figure 8F). Figure 8G displayed the confusion matrix of the model in local cohort. The accuracy, sensitivity, specificity, positive predictive value, and negative predictive value of the RF model in each cohort were shown in Table 5.

**Table 5:** The predictive performance of the random forest model in each cohort.

**Cohort**	**Accuracy**	**Sensitivity**	**Specificity**	**Positive predictive value**	**Negative predictive value**
GSE9210	1.000	1.000	1.000	1.000	1.000
GSE145467	0.900	0.833	1.000	1.000	0.800
Local cohort	0.725	0.800	0.680	0.600	0.850

## DISCUSSION

NOA includes changes in spermatogenesis caused by various hypothalamic and pituitary diseases, as well as primary spermatogenesis failure caused by different etiologies, and usually has a poor prognosis [21]. Such patients have no obvious signs of obstruction in the ultrasound examination of the reproductive system, but the obvious feature is that the patient's testicular volume is often small and cannot produce sperm or produce very few sperm. Patients with OA can often be diagnosed with ultrasound of the reproductive system, but some azoospermia patients exhibited both spermatogenic dysfunction and reproductive tract obstruction, which is known as mixed azoospermia. Therefore, imaging methods such as the ultrasound are far from sufficient to confirm the diagnosis of NOA. Many biomarkers associated with NOA have been discovered, such as follicle-stimulating hormone [22], serum inhibin B [23], and anti-Mullerian hormone [24], but more studies on the biomarkers are helpful for clinicians to achieve a more precise diagnosis.

In addition, from the perspective of pathogenesis, although many theories have been proposed to explain the pathogenesis of NOA, our understandings of the biological processes associated with NOA remains insufficient. Nowadays, the widespread applications of gene sequencing, especially scRNA-seq, have deepened the knowledge of NOA [25, 26]. For instance, Wang et al. had disclosed the unique role of autophagy homeostasis in the spermatogenesis of NOA cases through scRNA-seq analysis [27]. Liu et al. utilized scRNA-seq to detect the genetic change of ACE2 in testicular cells of normal and NOA patients, uncovering the possible mechanisms of how SARS-CoV-2 affected testicular cells [28]. These studies strongly demonstrated the usefulness of scRNA-seq for NOA’s mechanism detection. However, the scRNA-seq-based diagnostic model for NOA has not been reported.

Here, the scRNA-seq data of the testicular cells extracted from an NOA patient was analyzed. The cell markers, which were defined as the DEGs among different cell clusters, were used to construct a PPI network. GO and KEGG enrichment indicated the genes in the network were mainly involved in the cell cycle-related pathways, such as DNA replication, translational initiation, and regulation of G2/M transition of mitotic cell cycle. Subsequently, the Top 5 hub genes in the PPI network were chosen for diagnostic model development. GSE9210 and GSE145467 were utilized to construct and externally validate the predictive model, respectively, and a sum of 78 cases was enrolled, including 57 NOA and 21 OA patients. We also collected the seminal plasma samples of 20 OA and 20 NOA patients from The Third Affiliated Hospital of Southern Medical University, and detected the diagnostic efficacy of the RF model via RT-qPCR. Another important highlight of the research was that the random forest algorithm, a dimension reduction machine learning technique, was adopted for predictive model construction. The ROC analyses in the training cohort (AUC = 1.000), external validation cohort (AUC = 0.900), and local cohort (AUC = 0.725) demonstrated the feasibility and effectiveness of the strategy.

Some novel biomarkers for NOA were also screened. Ribosomal Protein S4 X-Linked (RPS4X) was essential for the formation of cytoplasmic ribosomes and participated in the initiation and progression of multiple diseases [29–31]. RPS4X acted as the strongest predictor in the random forest model with the highest Mean Decrease Accuracy and Mean Decrease Gini. ROCs also indicated that RPS4X was a promising diagnosis biomarker for NOA both in the training (AUC = 0.932) and external validation (AUC = 0.920) cohorts. RPS4X was significantly up-regulated in the iPS cells of testicular tissue isolated from the patient with NOA.

All the evidence suggested RPS4X played an important role in NOA. However, the association between RPS4X and NOA has never been reported, and how RPS4X regulated spermatogenesis of NOA patients remains unclear. Ribosomal Protein L36a (RPL36A) was also played an important role in the exertion of ribosomal function. The association of RPL36A with infertility has been reported. Selvaraju et al. has found RPL36A was up-regulated in the high-fertile bulls’ sperm, but the roles of RPL36A in human infertility are still unknown [32]. Overall, our findings helped to identify novel biomarkers, providing the possible cut-in for further elucidation of the mechanisms in NOA.

The limitations of the present study should be acknowledged. First, the research is retrospective, and a large-scale, multi-center, and prospective clinical trait would be beneficial to confirm the usefulness in clinical practice. Second, several novel biomarkers were identified, but their biological functions in NOA are unknown, and a series of experimental exploration ought to be conducted.

In this paper, we presented a random forest diagnosis model to distinguish NOA from OA, which was based on scRNA-seq analysis and externally validated, providing novel insights into the underlying mechanisms of NOA.

## Supplementary Material

Supplementary Figure 1

Supplementary Table 1

Supplementary Table 2
