# Supplementary material for: Construction and external validation of a 5-gene random forest model to diagnose non-obstructive azoospermia based on the single-cell RNA sequencing of testicular tissue
Source: Aging (Albany NY). 2021 Nov 4;13(21):24219–35. doi: 10.18632/aging.203675 (PMC8610122; doi:10.18632/aging.203675)
Supplement: Supplementary Figure 1 [file aging-13-203675-s001.pdf]

SUPPLEMENTARY FIGURE

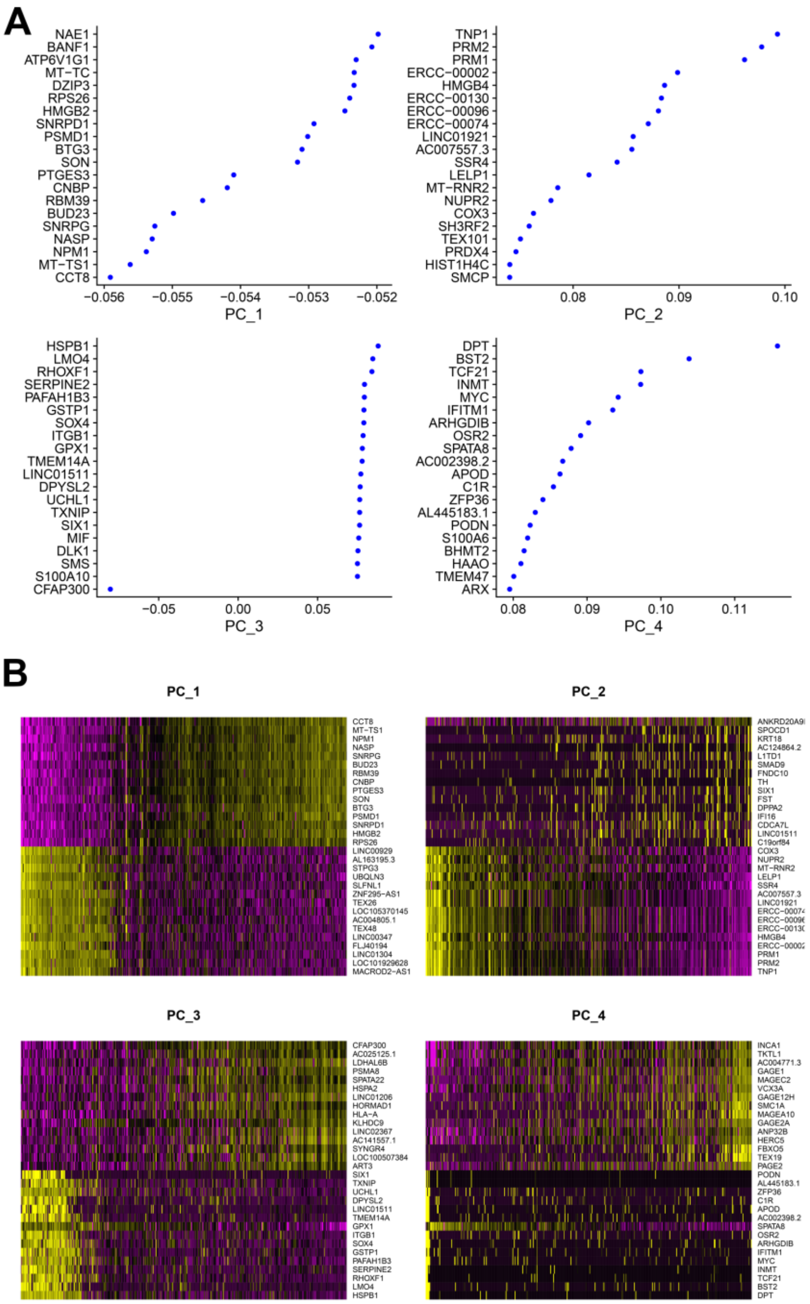

Supplementary Figure 1. The top 4 components and the correlated genes in PCA analysis. (**A**) The Top related genes to each principal component. (**B**) The heatmap indicating the expression level of the Top related genes. The colors ranging from purple to yellow represented the expression values from low to high.
