# Supplementary material for: Construction and external validation of a 5-gene random forest model to diagnose non-obstructive azoospermia based on the single-cell RNA sequencing of testicular tissue
Source: Aging (Albany NY). 2021 Nov 4;13(21):24219–35. doi: 10.18632/aging.203675 (PMC8610122; doi:10.18632/aging.203675)
Supplement: Supplementary Table 2 [file aging-13-203675-s003.pdf]

SUPPLEMENTARY TABLE

Supplementary Table 2. The analysis of the PPI network.

node_name	MCC	DMNC	MNC	Degree	EPC	BottleNeck	EcCentricity	Closeness	Radiality	Betweenness	Stress	ClusteringCoefficient
UGT1A10	1	0	1	1	1.429	1	0.06667	1	0.2	0	0	0
UGT1A1	1	0	1	1	1.429	1	0.06667	1	0.2	0	0	0
RPL36A	6	0.46346	3	3	4.331	1	0.12	7.05	1.87059	0	0	1
RPL39	6	0.46346	3	3	4.291	1	0.12	7.05	1.87059	0	0	1
RPL10	6	0.46346	3	3	4.346	1	0.12	7.05	1.87059	0	0	1
PRKCE	1	0	1	1	1.403	1	0.06667	1	0.2	0	0	0
PPP1CA	1	0	1	1	1.403	1	0.06667	1	0.2	0	0	0
LIN7B	1	0	1	1	1.747	1	0.06667	2	0.31111	0	0	0
HIP1	1	0	1	1	1.796	1	0.06667	2	0.31111	0	0	0
HIF1A	1	0	1	1	3.412	1	0.15	6.75	2.11765	0	0	0
PRIM1	1	0	1	1	3.035	1	0.12	6.35	1.87059	0	0	0
CLSPN	1	0	1	1	3.082	1	0.12	6.35	1.87059	0	0	0
GINS2	1	0	1	1	3.034	1	0.12	6.35	1.87059	0	0	0
FBXO5	2	0.30779	2	2	4.677	1	0.15	7.91667	2.29412	0	0	1
ESCO2	1	0	1	1	3.147	1	0.12	6.35	1.87059	0	0	0
CDC6	6	0.30779	2	6	5.521	5	0.15	9.91667	2.43529	116	116	0.06667
ITGB1	1	0	1	1	1.449	1	0.06667	1	0.2	0	0	0
CD9	1	0	1	1	1.449	1	0.06667	1	0.2	0	0	0
RPS4X	7	0.46346	3	4	5.06	4	0.15	9	2.36471	84	84	0.5
TUBA8	2	0.30779	2	2	4.384	1	0.15	7.5	2.18824	0	0	1
RGS6	1	0	1	1	3.558	1	0.15	7	2.15294	0	0	0
PDCL3	1	0	1	1	3.37	1	0.15	7	2.15294	0	0	0
TUBA4A	2	0.30779	2	2	4.562	1	0.15	7.5	2.18824	0	0	1
CCT8	7	0.30779	2	7	6.502	18	0.2	11.33333	2.71765	186	186	0.09524
PSMD1	5	0.30779	2	5	6.164	18	0.2	10.5	2.68235	152	152	0.2
BPI	2	0.30779	2	2	4.974	1	0.2	8.33333	2.43529	0	0	1
CHGB	1	0	1	1	1.43	1	0.06667	1	0.2	0	0	0
APLP2	1	0	1	1	1.43	1	0.06667	1	0.2	0	0	0
VAMP2	3	0	1	3	2.248	4	0.13333	3	0.4	6	6	0
AP1S2	1	0	1	1	1.747	1	0.06667	2	0.31111	0	0	0
